# Technological, Nutritional and Sensory Properties of an Innovative Gluten-Free Double-Layered Flat Bread Enriched with Amaranth Flour

**DOI:** 10.3390/foods10050920

**Published:** 2021-04-22

**Authors:** Antonio Piga, Paola Conte, Simonetta Fois, Pasquale Catzeddu, Alessandra Del Caro, Anna Maria Sanguinetti, Costantino Fadda

**Affiliations:** 1 Dipartimento di Agraria, Università degli Studi di Sassari, Viale Italia 39/A, 07100 Sassari, Italy; pigaa@uniss.it (A.P.); pconte@uniss.it (P.C.); asanguinetti@uniss.it (A.M.S.); cfadda@uniss.it (C.F.); 2 Porto Conte Ricerche Srl, Località Tramariglio, 07041 Alghero, Italy; fois@portocontericerche.it (S.F.); catzeddu@portocontericerche.it (P.C.)

**Keywords:** gluten-free flat bread, starch, sensory analysis, shelf life, texture

## Abstract

Celiac disease is increasing all over the world. In this context, most recent research in this area is addressing and attempting to improve the nutritional value and sensory characteristics of gluten-free (GF) food products and to enhance their technological properties. Here, amaranth flour was studied as a potential healthy ingredient for the development of an innovative GF flat bread. Starting from two different basic formulations (rice flour:corn starch and rice flour:tapioca starch, 50:50), the impact of partially replacing rice flour (6%) and starch (6%) with amaranth on the nutritional characteristics, polyphenol composition, textural, and sensory properties of the resulting GF flat breads was explored. The substitution with amaranth led to detrimental effects on the doughs’ viscometric properties, especially in the case of tapioca starch, but significantly improved the doughs’ textural properties. All the amaranth-enriched flat breads showed a better color and a significant increase in all polyphenols fractions but lower antioxidant activity. During bread storage for three days, a detrimental effect on both starch retrogradation, toughness, and extensibility properties were observed, especially when tapioca starch was used. Check-all-that-apply (CATA) sensory test results showed that the incorporation of amaranth increased yeast odor and yeast flavor perception and decreased the softness in mouth-only in tapioca-based samples. A better compromise among technological, nutritional, and sensory properties was achieved when amaranth flour was added to the basic rice and corn formulation.

## 1. Introduction

In recent years, the development and innovation of gluten-free (GF) products have gained considerable attention due to the increasing number of celiac patients [1]. Celiac disease (CD) is a chronic, immune-mediated enteropathy that damages the microvilli, which are tiny, hair-like projections present in the small intestine responsible for nutrient absorption [2]. The ingestion of the alcohol-soluble prolamin fractions of gluten proteins, as well as gliadin fraction from wheat, secalin from rye, hordein from barley, and avenin from oat, causes an autoimmune response resulting in chronic intestinal inflammation in celiac people [3]. The only treatment to overcome this disease is to follow a strict gluten-free diet [4], although new therapeutic agents as alternatives to the gluten-free diet have been under investigation for several years. Sparks et al. [5], in their review, summarized these novel therapeutics, like the use of digestive enzymes capable of digesting prolamins, to avoid the immune response in CD patients; treatment with peptide-binding agents, like the larazotide acetate, is able to inactivate gluten, and the use of vaccines is designed to induce immune tolerance to gluten. They also affirmed that these different therapies show some promise, even if their efficacy still needs to be demonstrated before considering them as an alternative to the gluten-free diet.

It is well known that the gluten-free diet shows many limitations from nutritional, technological and sensory points of view. In fact, the GF diet is low in protein, fiber, iron, B group vitamins and folate, and it is rich in fat and carbohydrates [6]. This is the reason why most of the recent research is focused on fortifying the nutritional quality of GF foods, in particular bread, using nutrient-dense alternative flours, such as quinoa or amaranth, and to explore the role of functional ingredients (such as hydrocolloids, proteins or natural food supplements) capable of improving food’s physico-chemical, technological and sensory properties [7]. More recently, novel products have been developed using GF grains [3]. Among these, amaranth (*Amaranthus* spp.) has been rediscovered in the last years due to its high nutritional and commercial value. Amaranth is very rich in vitamins and minerals, and its protein content is higher than other cereal grains. Amaranth proteins are also rich in essential aminoacids like methionine and lysine. In addition, amaranth contains phenolic compounds like caffeic acid, p-hydroxybenzoic acid and ferulic acid, whose biological and physiological functions are very well-known [8]. Few papers are present in the literature dealing with the impact of amaranth flour incorporation on dough rheology and bread quality [9,10]. In both studies it is reported that the addition of amaranth to GF foods increases water holding capacity and enhances the viscoelastic properties and the content of micronutrients. For this reason, the effect of this underutilized pseudocereal on the rheology, functionality and sensory acceptability of dough and bread quality needs to be investigated further.

Flat breads (FB) are the oldest and most widely known bread type (tortilla, focaccia, baladi, pita, chapati, roti, kisra), produced worldwide in regions like Scandinavia, South Europe, North Africa, the Middle East, East and part of China and Central America. These can be obtained by using flours containing gluten (wheat, barley, rye) or made from GF grains (corn, sorghum, teff) [11]. In some Mediterranean countries, semolina from durum wheat (*Triticum turgidum* subsp. *durum* L.) is used to produce not only pasta and cous cous but also leavened bread [12]. In fact, two-layered flat breads are very popular in the Middle East, Arab countries, and in Sardinia [13], where a durum wheat flat bread called “spianata” is produced. It is well known that durum wheat induces an immune response in CD patients. This is the reason why, in the past few years, throughout the world, the demand both for flat bread [14] and for gluten-free products is increasing [15]. Many technological characteristics of flour as well as water absorption, gluten quality, starch gelatinization, and moisture content are very important to produce flat bread with balanced visco-elastic properties and are fundamental to the sheeting and moulding steps during processing. As is well known, gluten is essential to obtain a strong protein network able to develop bread with good volume and texture properties [16]. Furthermore, the technological importance of the gluten permits wheat doughs to develop important characteristics such as adequate dough machinability, water binding capacity, viscosity, and elasticity. Therefore, the main strategy for the replacement of gluten is to use functional ingredients able to mimic gluten’s properties. Several kinds of additives have been studied to improve quality of GF breads, and in particular the hydrocolloids and gums have been the object of many studies that have confirmed their capacity to act as thickening and gelling agents, thus improving GF food quality and shelf life from a technological and sensory point of view [17,18]. Proteins are also used in the GF food industry to improve texture and to fortify GF foods from a nutritional point of view [19,20]. In addition, the role of enzymes has been explored, evidencing their great importance as functional ingredients [21]. In particular, amylase showed a great ability to increase bread volume [22]. Despite the vastity of ingredients studied to reach an appropriate balance between the elastic and viscous properties of GF doughs, starch remains the primary structural element able to determine the quality parameters of the final breads [15,23]. Nowadays, several flours derived from alternative grains, legumes, and seeds have been assessed and used for improving the nutritional value and palatability of GF bread formulation [24]. In the literature, many papers deal with the impact on the viscoelastic properties of GF dough of different flours and starches, but very few papers [9,17,25] have explored the influence of different starches (corn and tapioca) and the partial substitution of rice flour and starch with an alternative flour, as well as amaranth, on the production and quality of GF flat breads. The object of this study was the development of an innovative GF flat bread using the traditional recipe of the “spianata” considering the high incidence of the celiac disease in the Sardinia region (450 cases per 100,000 people) [26].

Thus, the aim of this study was to explore the effect of two different starches, namely corn and tapioca starch, singly blended with rice flour, and the impact of their partial substitution with amaranth flour on the nutritional characteristics, polyphenol composition and textural and sensory properties of a GF double layered flat bread, the so-called “spianata”.

## 2. Materials and Methods

### 2.1. Raw Materials

Rice flour (R), corn starch (C), tapioca starch (T) and *Psyllium* fiber (*P*) were from Chimab (Campodarsego, Italy), while amaranth flour (A) was from Molini Bongiovanni S.p.A.–Cambiano (TO, Italy). The proximate chemical and nutritional composition of the main ingredients were provided by the manufacturers and reported in Table 1. Baker’s yeast was from Casteggio Lieviti (Pavia, Italy) and salt from Lisal (Cagliari, Italy).

### 2.2. Breadmaking

A double layered GF flat bread was produced following the traditional recipe of spianata bread, which is further referred to as GF spianata. Four different combinations of flours and starches were developed: (1) RC, which is 50% R plus 50% C; (2) RT, which is 50% R plus 50% T; (3) RCA, which is 44% R, 44% C, and 12% A; and (4) RTA, which is 44% R, 44% T, and 12% A. Salt (1%), baker’s yeast (1.5%), psyllium fiber (3%) and water (65%) were added to the formulations, flour basis. All the ingredients were mixed in fork mixer Arca50 (Sottoriva Spa, Marano Vicentino, Italy) for 6 min. Then, the dough was manually kneaded to obtain three cohesive pieces, which were gently passed through a mechanical dough sheeter (Manomat 2000, Rondo Srl, Schio, Italy) with an initial gap of 25 mm. Each dough piece was sheeted 1 time, progressively reducing the gap between the rollers until a thickness of 2 mm had been reached. The sheeted doughs were then shaped using a 22-cm diameter circular mold, placed in an aluminum tray and covered with plastic film to avoid water loss. Then, the doughs were rested in a Levcontrol (LC) plus Tecnomac fermentation chamber (Castelmac Spa, Castelfranco Veneto, Italy) held at 28 °C and 72% relative humidity for 15 min. The breads were baked in an electrical tunnel oven (Superabo/60 F.S, Rinaldi Superforni Srl, Massa, Italy) at high temperature (470 °C for 20 s), with the aim to quickly vaporize the moisture within the dough and cause it to puff up.

**Figure 1:**
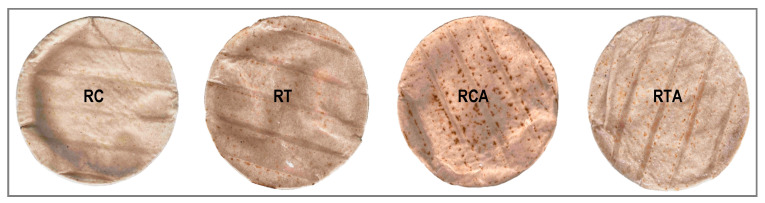
Images of the four experimental GF flat breads.

GF spianata was then set aside at room temperature until cool and covered with cotton fabric to avoid hardening (Figure 1). The bread was packaged using a multilayer (EVOH⁄OPET⁄ PE) gas and water barrier film of 54 µm thickness (EOM 360B, Sealed Air, USA) with the following gas transmission rates: O_2_, 4 cm^3^ m^−2^ 24 h^−1^ bar^−1^ at 23 °C; CO_2_, 13 cm^3^ m^−2^ 24 h^−1^ bar^−1^ at 23 °C; water vapor, 9 g m^−2^ 24 h^−1^ at 38 °C. A sachet of an iron oxide-based oxygen absorber (ATCO FT 210, Standa Industries, France) was placed inside the trays to avoid mold development. The bread was then stored under controlled conditions (20 ± 0.5 °C and 55 ± 1% relative humidity). Four randomly selected GF spianatas were analyzed using both NIR FT-Raman spectroscopy and texture analysis after 0, 1, 2, and 3 days. Twelve GF spianatas for each formulation, just after 4 h from the moment they were removed from the oven, were stored in the freezer at a temperature of −20 °C until the time of sensory analysis, due to practice requirements.

### 2.3. Doughs Viscometric and Textural Properties

To gain information about the viscometric properties of the four GF doughs produced, RC, RT, RCA and RTA, pasting profiles were obtained using a Rapid Visco Analyser (RVA-4, Newport Scientific, Warriewood, Australia) according to the International Association of Cereal Science and Technology (ICC) Standard method 162 [27]. The pasting temperature (°C), peak time (when peak viscosity occurred; min), peak viscosity (maximum hot paste viscosity), breakdown (peak viscosity minus holding strength or minimum hot paste viscosity), setback (final viscosity minus holding strength), and final viscosity (end of the test after cooling to 50 °C and holding at this temperature) were calculated. All the viscosity parameters are expressed in mPa s. Dough samples were prepared by dispersing 3.5 g of each flour/starch formulation, as reported in the paragraph 2.2, in 25 mL of distilled water into an aluminum canister. Doughs were then subjected to heating and cooling following these steps: holding at 50 °C for 1 min, gradual heating to 95 °C, holding at 95 °C for 2 min 30 s, cooling to 50 °C, and lastly holding at 50 °C for 2 min.

Doughs were also subjected to texture analysis through the application of stress-relaxation and penetration tests [28] using a TA.XT2i Texture Analyzer (Stable Micro System Ltd., Godalming, Surrey, UK) equipped with a 25 kg load cell. Tests were conducted in triplicate on the doughs. The software Texture Expert Exceed was used to process the data obtained. The stress-relaxation test was applied on dough discs prepared in the alveograph machine (AACC Approved Method 54-30.02A) [29]. The stress relaxation (% SR) was calculated as the force registered after 35 s, divided by the maximum registered force in percentage [30]; % SR is considered the best parameter to interpret foods’ viscoelastic behavior. The resistance to penetration was assessed according to [31]. To assess this parameter, a puncture test was performed by using a probe with a diameter of 5.5 mm that penetrated the sample at 15 mm/s. Data were expressed in N.

### 2.4. GF Spianatas Quality

#### 2.4.1. Proximate Chemical Composition

The moisture, ash, and protein content of GF spianatas were determined according to the Official Standard Methods AACC 44-15.02, 08-01.01 and 46-12.01, respectively [29]. The total lipid content was obtained by gravimetric analysis using the Soxhlet apparatus and petroleum ether as a solvent during a 6-h period. Total carbohydrates were calculated by difference. Analyses were conducted in triplicate.

#### 2.4.2. Analyses of Polyphenolic Fractions and Antioxidant Activity

Phenolic fractions were determined following the procedures reported in [32]. Of the ground GF flat bread sample, 1 g was subjected to extraction at room temperature, two times, with 4 mL of a solution of 37% hydrochloric acid/methanol/water (1/80/10, *v/v/v*). The two collected supernatants, after filtration, were used to analyze the soluble phenolic fraction. The remaining residues were digested in a shaking water bath, at a temperature of 85 °C for 20 h with 5 mL of a solution composed of methanol and sulphuric acid concentrate (10:1, *v/v*) to obtain the insoluble phenolic fraction. The two extracts were analyzed by spectrophotometry (Spectrophotometer Hewlett Packard, Palo Alto, California) at a wavelenght of 750 nm, following the Folin-Ciocalteau method reported in [33]. Then, the bioaccessible polyphenol fraction was evaluated using an “in vitro” enzymatic digestion adapted for bread [34]. Of the ground GF flat bread sample, 1 g was incubated with 0.5 mL of pepsin and 10 mL of distilled water and put in a shaking water bath for 1 h at 37 °C. Then, pH was adjusted to 2 with 5 M of hydrochloric acid solution. The gastric digestion was interrupted by adding sodium hydrogen carbonate (1 M) to the sample until it reached the pH of 7.2. Then, to simulate intestinal digestion, 2.5 mL of bile/pancreatin solution and 2.5 mL of sodium chloride/potassium chloride solution were added to the sample. After an addition of trichloroacetic acid (20% *v/v*) to remove proteins, 2 mL of the digested extracts were supplemented with 0.5 mL of Folin-Ciocalteau reagent, 10 mL of sodium carbonate (7.5%), and adjusted to 25 mL with distilled water. The mixture was incubated in the dark for 1 h at room temperature, and the absorbance was measured at 750 nm. All the polyphenols fractions were expressed as mg of gallic acid equivalent per 100 g of bread dry matter, using calibration curves made with gallic acid (mean of three replicates).

The antioxidant activity was evaluated using the DPPH (2,2-diphenyl-1-picrylhydrazyl) method [32] with slight modifications. In brief, 0.3 mL of organic extracts were added to 2.7 mL of 0.0634 μmol/mL DPPH methanol solution. The absorbance was read at 515 nm after 1 min and every 5–10 min until reaching the plateau (60 min). Flat bread samples were analyzed in triplicate, and plots of μmol DPPH vs. time (min) were drawn. The antioxidant activity (AA) was calculated using the following equation:

AA = [(DPPH initial−DPPH plateau) × 100]/DPPH initial.(1)

#### 2.4.3. Texture Analysis

Texture measurements (“Tortilla test”) on the flat bread spianatas were made at zero, one, two and three days of storage, using the TA.XT2i Texture Analyzer (Stable Micro Systems Ltd., Godalming, Surrey, UK) equipped with a 25-kg load cell, and analyzed using version 2.64 Texture Expert Exceed software. The fixture used was the HDP/TPB (tortilla/pastry burst rig), which permits measurements of extension and elasticity on laminated pastry and tortilla dough. The measurements were recorded as a force-deformation curve. Variables recorded were the maximum force (N) and distance (mm) needed to compress the tortilla until its rupture (note that the greater the distance at the break, the more extensible is the sample). The break force and distance to break values are used as an indication of ‘resistance to deformation’ or ‘toughness’ and ‘extensibility’, respectively. Four GF spianatas for each formulation were analyzed.

#### 2.4.4. Color Analysis

GF spianata color was determined at five points, three external and two central, on the surface of each GF spianata, with a tristimulus colorimeter (Chromameter-2 Reflectance, Minolta, Osaka, Japan) equipped with a CR-300 measuring head. Hunter Space color was used to express colorimetric data as L, lightness, from 0 (black) to 100 (white), *a* coordinate from redness (+) to greenness (−), and *b* coordinate from yellowness (+) to blueness (−). Then, the Browning index was calculated as 100-L [35].

#### 2.4.5. Starch Retrogradation

For NIR FT-Raman spectroscopy analysis, a small piece of bread (~5 g) was removed from the center of the slice. Measurements were performed using back-scattering geometry with a Bruker RAM II FT-Raman module coupled to a Bruker Vertex 70v interferometer, as previously reported by [36]. The laser excitation wavelength was 1064 nm, and each spectrum was acquired by averaging 128 interferograms at a resolution of 4 cm^−1^ in the range of 250–3500 cm^−1^ with a Blackmann-Harris 3-Term apodization function to more precisely determine the peak position of the pyranose ring breathing band that peaked at ~480 cm^−1^; this single band was selected by considering the spectral range of 460–510 cm^−1^. As residual fluorescence was present, following baseline correction, the band intensity was normalized in this selected range. Finally, post-zero filling was applied to the 460–510 cm^−1^ spectral range, with a zero-filling factor of 64, providing a very accurate band profile. With this spectral pre-processing and using the peak-picking function of the Bruker OPUS 6.5 software, the peak position was obtained with an accuracy of 0.01 cm^−1^, as certified by Bruker Corporation (Billerica, MA, USA).

#### 2.4.6. Consumer Study with CATA Test

A total of sixty consumers (all non-celiac), aged between 20 and 61 years, were randomly recruited at the University of Sassari, Department of Agricultural Sciences. Participants were approximately 50% female and 50% male and were selected for their regular consumption of flat bread. Their participation was voluntary. The four GF spianatas prepared for the sensory analysis were defrosted and heated for one minute at a temperature of 180 °C before being served. GF flat bread were codified with a three-digit code number and presented, one at a time, to the consumers, using a randomized and balanced order to avoid carry-over effects. Again, as suggested by [37], the order of presentation of CATA attributes was balanced between and among the consumers. Water was provided between the samples.

Consumers were asked to taste GF flat bread and to evaluate on a structured 9-point hedonic scale (1 = ‘‘dislike extremely”, 9 = ‘‘like extremely”) the overall liking for each sample. Then they were asked to answer a check-all-that-apply (CATA) question by selecting the attributes they considered most appropriate to describe the GF flat breads. The attributes list was set before the test by using previous literature on flat bread sensory description [17,25]. The sensory attributes were as follows: good color; soft to touch; sweet; perfect as a snack; chewable; yeast odor; good for nutrition; traditional bread odor; adhesive; good as a side dish; yeast flavor; healthy; cohesive; good for diet; dry; soft in mouth; off-odor; salty.

### 2.5. Statistical Analysis

Data were analyzed with a one-way analysis of variance (ANOVA) with the Statistica v10.0 software (StatSoft, Inc., Tulsa, OK, USA). Fisher’s least significant differences (LSD) test was applied to assess the difference between each pair of means, when significant. Sensory data were analyzed using XLSTAT for Windows (Version 2020.1.2, Addinsoft, Paris, France). To identify differences in overall liking attribute among samples a one-way ANOVA was performed, considering as fixed source of variation the samples and the consumers as random. Cochran’s Q test, followed by the McNemar (Bonferroni) multiple pairwise comparison test (*p* < 0.05), was applied to the CATA data to find significant differences between samples for each term present in the CATA questionnaire. At last, correspondence analysis (CA) was performed on the frequency table to obtain a bi-dimensional representation of the samples and the relationship between the chosen attributes and the flat bread samples.

## 3. Results and Discussion

### 3.1. Doughs Viscometric Parameters and Textural Properties

Data on the GF doughs’ viscometric properties are reported in Table 2. It is well known that starches from different origins can influence bread structure and texture. The role of starch is to bind water and form a gas-permeable structure that can modify dough rheology [23]. In fact, the gelatinization of starch granules permits them to trap air bubbles, causing a modification of the structure with higher volume and more softness. However, the behavior of starches can be different depending on their origin, their amylose/amylopectin content, and their pasting and gelling properties. In this study, it can be observed that the two experimental starches behaved in a different way. In fact, as reported in Table 2, while the RC sample showed breakdown values significantly lower, but values of final viscosity significantly higher, than those observed in the RT doughs, no significant differences were observed in terms of peak viscosity among the two basic formulations. In the case of RT, however, the time to reach the peak was shorter, suggesting that tapioca starch may promote granule swelling during the heating cycle. Similar conclusions were previously observed in both tapioca and potato starch by [38], who reported that root starches gelatinize faster and at lower temperatures than cereal starches, probably due to their lower degree of crystallinity. In spite of similar peak viscosity values, the replacement of corn starch with tapioca starch also led to a more pronounced drop in the viscosity of the system, presumably as a consequence of the higher bonding forces within the granules, which caused a more severe breakdown and, in turn, a lower stability of the system [39]. Moreover, during the cooling stage, the values of setback and the final viscosity of the RT samples were found to be significantly lower than those of the RC doughs, suggesting that tapioca starch may have a low tendency to promote starch retrogradation. Similar findings were observed by [40], who reported that tapioca starch suspensions exhibit low pasting temperatures and final viscosity values much lower than their peak viscosities due to significant granule breakdown. The partial replacement of both rice flour and starches with amaranth flour significantly lowered the viscometric properties of the resulting GF doughs, this effect being more pronounced in the case of RTA. During gelatinization and pasting, values of both the peak viscosity and the breakdown of the amaranth-enriched samples decreased, with respect to the basic formulations, in a similar manner in the presence of both corn and tapioca starch, probably as a consequence of water competition that hinders the starch swelling, or because swelling of one starch is inhibited by the other [40,41]. Conversely, during the cooling phase, an increase in both setback and final viscosity was observed only in the sample RTA, indicating that the substitution of tapioca starch rather than corn starch significantly increased the extent of retrogradation. Generally, as the system cools, a transition from a viscous liquid to a gel containing gelatinized starch granules takes place, causing an increase in the viscosity of the system primarily due to the retrogradation of soluble amylose molecules. In the present study, however, considering that amaranth starch is usually characterized by low content of amylose or is waxy, a possible explanation of such behavior could be related to an increase in the interactions between the amylose leached from tapioca starch and the long chain amylopectin of amaranth, which generate a synergistic effect on the final viscosity and, thus, on starch retrogradation [40,42].

With reference to the % SR data (Table 3), a decrease in the GF formulations that were amaranth enriched was observed, confirming that the presence of this pseudocereal confers a lower elasticity to the GF doughs. Data of resistance to penetration showed the same behavior for RC and RT doughs, which showed a tendency to be softer when amaranth was added to the dough, confirming the data reported in [9] on the increase of softness in doughs supplemented with amaranth at percentages ranging from 20–50%.

### 3.2. GF Spianatas Quality

#### 3.2.1. GF Spianatas Physico-Chemical Analyses

As can be seen from Table 4, the addition of amaranth to the mixture of RC and RT increased the nutritional bread value. The protein and ash content of RCA and RTA bread samples were significantly higher than the formulations without amaranth, confirming the role of this pseudocereal as an ingredient able to improve the nutritional value of cereal-based foods [9,43].

The color measurements are reported in Table 5. No significant differences were found for the L parameter, although the RC sample, when supplemented with amaranth, tended to become darker. Moreover, an increase in redness and yellowness was observed in RCA. GF spianatas made with tapioca and amaranth showed a significant increase in yellowness, but the redness parameter was not significant. Our findings are similar to those reported in the literature [43,44]. The Browning index was not significantly different among the four GF spianatas.

#### 3.2.2. Polyphenolic Fractions and Antioxidant Activity of GF Spianatas

Data on the polyphenols content are reported in Table 6.

**Figure 2:**
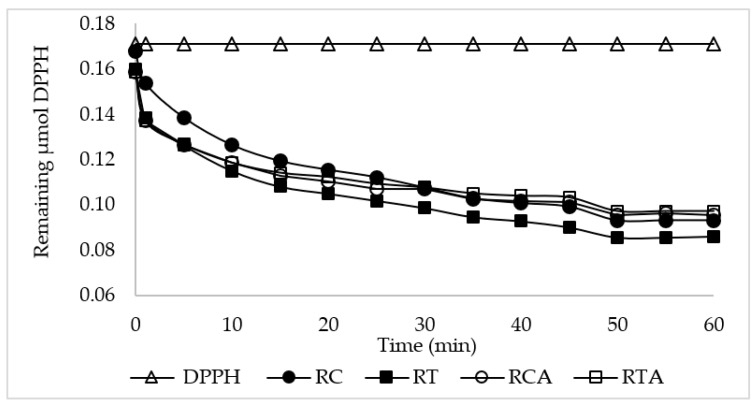
Time evolution of the DPPH curves in methanol of organic extracts from FB samples.

The addition of amaranth flour caused a significant increase in all the polyphenol fractions for both GF formulations. Moreover, it can be observed that the content of the insoluble polyphenols was higher than the soluble fraction. These findings confirmed what is reported in the literature on cereal and cereal-based baked foods [32,45]. To establish the biological activity of these fractions, the bioaccessible fraction was also determined. Data showed a significant increase in polyphenols’ bioavailability for FB supplemented with amaranth flour, with respect to the formulations RC and RT. The major increment, 23%, was observed for the RC formulation, whereas for the RT formulation, the increment was only 9.7% (Figure 2). However, these are the first results obtained on flat breads with reference to the polyphenolic fractions, and no comparison with data reported in literature has been possible.

With reference to antioxidant activity, our finding showed a decrease in this parameter in GF formulations enriched with amaranth flour, confirming data reported in the literature [44,46], particularly those reporting low substitution percentages. Despite this, the mechanism of this decrease still needs to be explored.

#### 3.2.3. Texture Analysis of GF Spianatas during Storage

**Figure 3:**
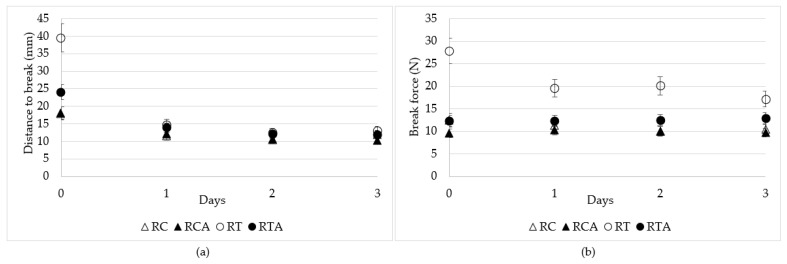
Parameters of “Tortilla test”: (**a**) break distance and (**b**) break force in FB samples over a storage period of three days.

Flat bread storage was monitored over a period of three days using a texture analyzer, as reported in Section 2.4.3. The distance to break and the break force, which were measured using the tortilla test over time, did not show any significant change, with the exception of the distance to break in the RT formulation during the first 24 h (Figure 3a,b). The formulation RT was characterized by a greater extensibility than the RC one, thus resulting in a better quality with which to produce spianata flat bread. The addition of amaranth flour had a detrimental effect on spianata quality in the RT formulation and no effect in the RC formulation, either in the distance to break or in the break force value. After the first 24 h, the behavior of the different formulations was very similar, apart for the break force value (toughness), which was higher during all storage times in the RT formulation.

#### 3.2.4. Starch Retrogradation

As reported by [36], the Raman spectrum of starch is characterized by typical bands, among which the spectral ranges of 400–640 cm^−1^ can be attributed to the skeletal modes of the pyranose ring, which show a typical strong band at ~480 cm^−1^. This band is a characteristic starch signal that is known to be sensitive to starch crystallinity and conformation [47]. The changes in this Raman band have been shown to be related to starch retrogradation over time [36], and retrogradation of starch is one of the main phenomena that govern bread staling. The Raman frequency of this band shifted to lower frequencies over time, as observed in our data.

**Figure 4:**
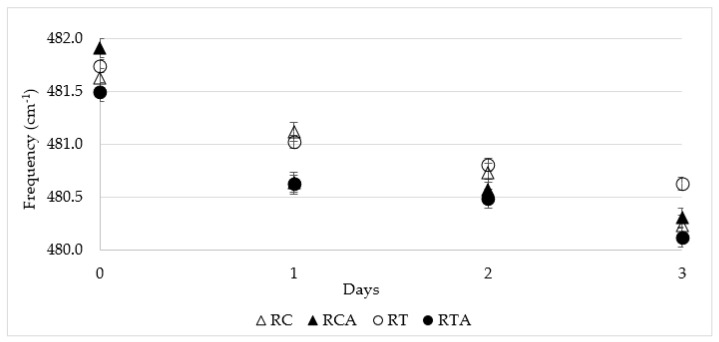
Variations in peak frequency of the Raman band at ~480 cm^−1^ during storage.

As shown in the Figure 4, most of the total frequency shift occurred over the first 24 h of storage. The addition of amaranth flour had a contradictory effect on retrogradation level of starch at time zero (the day of baking), while it showed a clear negative effect after 24 h of storage in both RCA and RTA formulations. As the bread moisture did not change at all over this storage period (data not shown), the observed changes in the starch properties can be referred to as starch retrogradation. Therefore, it is likely that the high fiber content of amaranth determined a migration of water from starch, thus causing an increase in retrogradation.

### 3.3. Sensory Evaluation

Among the 18 attributes chosen, only a few of them, namely soft to touch, yeast odor, yeast flavor, adhesive, cohesive, soft in mouth and dry, significantly differed among the experimental flat bread samples (Table 7).

It can be noted that the use of amaranth flour did not significantly modify the sensory characteristics of the formulation with corn but increased the yeast odor and flavor perception in the RTA sample. The softness in mouth attribute was significantly different in the formulation with tapioca, showing a decrease when amaranth flour was added.

**Figure 5:**
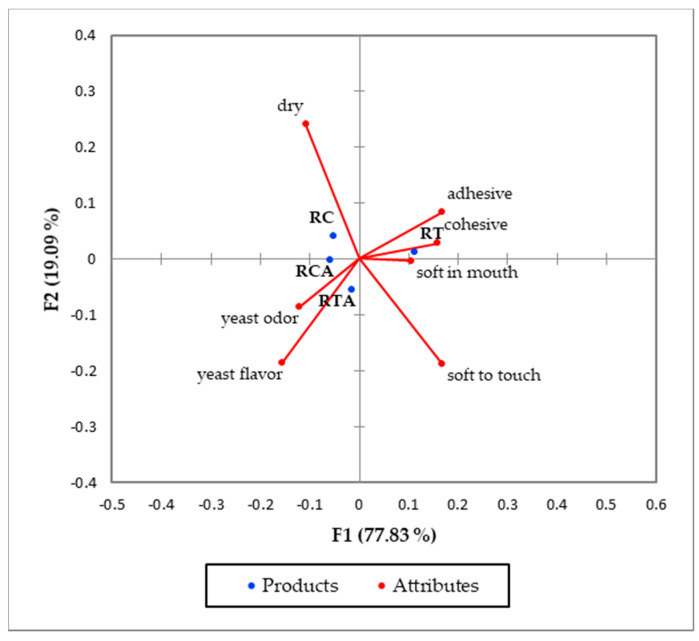
Correspondence Analysis of flat breads with different formulations.

The results of the Correspondence Analysis are reported in Figure 5.

The 96.9% of total inertia was explained by the two factors. The RT flat bread sample resulted more adhesive, cohesive, soft in mouth and to touch than the other three spianata samples. RC was considered the driest sample by the consumers, while the two amaranth-enriched spianatas exhibited a high intensity of yeast odor and flavor.

One-way ANOVA applied on overall liking data showed no significant differences among the four samples. In particular, on a scale from 1 to 9, overall liking mean values were as follows: 5.8, 6.1, 5.9, and 5.8 for RC, RT, RCA, and RTA, respectively.

The opinions of consumers were in the range of “neither like nor dislike” to “like slightly”. It must be considered that appreciation for gluten-free bread is always very low. Moreover, the use of increasing percentages of amaranth flour tends to negatively affect the consumer sensory perception. In fact, in the literature is reported that a percentage of amaranth substitution of 15–20% causes a slight depreciation of the physical and sensory characteristics of bread [9,43,48], which increases with higher percentages. This is because amaranth gives the bread an unusual nutty aroma, which, when percentages are higher than 20%, lead the consumer to reject it.

## 4. Conclusions

Finding new formulations for GF products with ideal nutritional properties is a big challenge for researchers. In this study, the impact of the partial replacement of rice flour and starch (corn and tapioca) with amaranth flour, an underutilized pseudocereal, on the overall quality of gluten-free double-layered flat breads was evaluated. Data obtained from the doughs’ pasting profiles evidenced that tapioca starch had the capacity to promote granule swelling during the heating cycle, as well as a low tendency to promote starch retrogradation during the cooling stage. The partial substitution with amaranth tended to increase the extent of retrogradation only in the sample with tapioca. Moreover, the presence of amaranth affected dough texture parameters, decreasing dough elasticity and increasing softness in both formulations, corn and tapioca. With reference to the GF flat breads quality, apart from an increase in protein and ash content, amaranth substitution caused an increase of all the polyphenol fractions, especially the bioaccessible fraction. Conversely, an unexpected decrease in the antioxidant activity % was found, but the reasons need to be explored more. Referring to the color changes, the GF flat bread with tapioca and amaranth showed a significant increase in yellowness. During storage, the partial substitution of tapioca starch with amaranth flour increased the break force value at all storage times. This detrimental effect was also confirmed by the analysis of Raman spectra, which evidenced an increase in starch retrogradation for both amaranth-enriched formulations over time. Finally, CATA results evidenced the role of amaranth in increasing yeast odor and yeast flavor and in decreasing the perception of softness in mouth when amaranth was added to the bread with tapioca. The overall liking was not significant different among the GF flat breads, probably due to the substitution percentage not exceeding 20%, which is considered a percentage of rejection for consumers. In conclusion, the supplementation of rice corn basic formulation with amaranth flour led to a better compromise among the technological, nutritional, and sensory properties of the resulting flat breads. However, further studies are needed to overcome some of the detrimental effects, especially the higher extent of starch retrogradation and the lower antioxidant activity observed in the present work.

## Figures and Tables

**Table 1:** Proximate chemical and nutritional composition of gluten-free ingredients (g per 100 g ingredient, as is).

Ingredients	R	C	T	A	*P*
Protein	7.1	0.3	0.5	14.5	2.5
Carbohydrate	76.5	88	86	51	4
Lipid	1.3	0	0.5	6.5	0.5
Fiber	0.2	0.0	0.5	15	81 *
Moisture	14	12	12.6	14.5	10
Ash	0.8	0	0.2	2.4	2

R: Rice flour, C: corn starch, T: tapioca starch and P: *Psyllium* fiber. * 44 soluble fiber, 36 insoluble fiber.

**Table 2:** Viscometric properties of GF doughs.

Samples	Parameters					
	Peak Viscosity (mPa·s)	Breakdown (mPa·s)	Setback (mPa·s)	Final Viscosity (mPa·s)	Peak Time (min)	Pasting Temperature (°C)
RC	4100 ± 163a *	1026 ± 66c	2317 ± 172a	5391 ± 261a	5.5 ± 0.2b	72 ± 7 ^ns^
RT	4170 ± 71a	1752 ± 21a	1086 ± 96d	3504 ± 47c	5.1 ± 0.1c	67 ± 10
RCA	3701 ± 70b	634 ± 5d	2038 ± 74b	5105 ± 31a	6.0 ± 0.0a	77 ± 0
RTA	3838 ± 45b	1196 ± 145b	1788 ± 41c	4430 ± 206b	5.4 ± 0.0b	73 ± 0

* Different letters within the column mean significant differences among the dough samples according to LSD test (*p* < 0.05). ^ns^ not significant.

**Table 3:** Mean values and standard deviations of dough texture parameters obtained through the application of the stress-relaxation and penetration tests.

Dough Samples	Stress Relaxation (%)	Resistance to Penetration (N)
RC	45.39 ± 0.62a *	0.599 ± 0.002b
RT	43.35 ± 0.33a	0.725 ± 0.022a
RCA	37.75 ± 0.35b	0.462 ± 0.018c
RTA	37.10 ± 0.77b	0.608 ± 0.049b

* Different letters within the column mean significant differences among the dough samples according to LSD test (*p* < 0.05).

**Table 4:** Proximate chemical composition of FB samples (g/100 g d.m.).

FB Samples	Moisture	Lipid	Ash	Protein	Total Carbohydrate
RC	32.3 ± 0.0c *	0.06 ± 0.02c	1.85 ± 0.02d	4.23 ± 0.21b	61.6 ± 0.1a
RT	33.7 ± 0.1b	0.08 ± 0.01bc	1.96 ± 0.06c	4.03 ± 0.01b	60.2 ± 0.0b
RCA	35.0 ± 0.1a	0.18 ± 0.03a	2.22 ± 0.03b	5.86 ± 0.07a	56.8 ± 0.1c
RTA	31.9 ± 0.1d	0.14 ± 0.01ab	2.33 ± 0.00a	5.69 ± 0.03a	60.0 ± 0.0b

* Different letters within the column mean significant differences among the dough samples according to LSD test (*p* < 0.05); d.m.: dry matter.

**Table 5:** Color parameters of FB samples.

FB Samples	L	a	b	Browning Index
RC	75.04 ± 2.05a *	−0.19 ± 0.14b	5.77 ± 0.12c	24.96 ± 2.05a *
RT	73.78 ± 1.47a	−0.05 ± 0.07b	5.84 ± 0.07c	26.22 ± 1.47a
RCA	72.71 ± 2.47a	0.96 ± 0.17a	9.66 ± 0.29a	27.29 ± 2.47a
RTA	74.24 ± 0.91a	−0.05 ± 0.43b	8.30 ± 0.24b	25.76 ± 0.91a

* Different letters within the column mean significant differences among the dough samples according to LSD test (*p* < 0.05).

**Table 6:** Mean values and standard deviations of polyphenol fraction and antioxidant activity % of FB samples.

FB Samples	Polyphenol Fractions (mg GAE */100 g Fresh Bread)				Antioxidant Activity % **
	**Soluble**	**Insoluble**	**Total**	**Bioaccessible**	
RC	23.3 ± 1.1d	80.6 ± 6.1b	104.0 ± 7.2b	62.1 ± 2c	44.5a
RT	30.9 ± 4.8c	60.3 ± 3.0c	91.2 ± 2.0c	71.6 ± 1b	46.3a
RCA	49.8 ± 1.2a	88.3 ± 5.1ab	138.1 ± 4.5a	80.8 ± 1a	39.9b
RTA	38.3 ± 0.6b	91.6 ± 4.7a	129.8 ± 4.2a	79.3 ± 1a	37.2b

* GAE: gallic acid equivalent. Different letters within the column mean significant differences among the FB samples according to LSD test (*p* < 0.05). ** Corresponding to 36 mg freeze-dried bread that consumed these percentages when 0.17 micromoles of DPPH are available to react.

**Table 7:** Attributes significantly different in Cochran’s Q test analysis (*p* < 0.05).

CATA Attributes	*p*-Values	RC	RT	RCA	RTA
Soft to touch	0.000	0.054b ^1^	0.339a	0.089b	0.214ab
Yeast odor	0.023	0.589ab	0.339b	0.482ab	0.607a
yeast flavor	0.008	0.304ab	0.143b	0.393a	0.492a
adhesive	0.000	0.107ab	0.304a	0.036b	0.089ab
cohesive	0.000	0.107b	0.357a	0.089b	0.125ab
Soft in mouth	0.029	0.036b	0.161a	0.054b	0.054b
dry	0.000	0.768a	0.446b	0.589ab	0.357b

^1^ Values with the same letter do not differ significantly from each other according to the McNemar (Bonferroni) multiple pair-wise comparison test (*p* < 0.05).

## Data Availability

Not applicable.
